# State-Level Flavored E-Cigarette Bans and Initiation Rates Among Youths and Adults

**DOI:** 10.1001/jamanetworkopen.2025.51744

**Published:** 2026-01-05

**Authors:** Meng-Yun Lin, Lindsey I. Abdelfattah, Amresh D. Hanchate, Erin L. Sutfin, Rachel L. Denlinger-Apte

**Affiliations:** 1 Department of Social Sciences and Health Policy, Wake Forest University School of Medicine, Winston-Salem, North Carolina

## Abstract

**Importance:**

The availability of flavored e-cigarettes contributes to adolescent and young adult e-cigarette initiation, exposing users to nicotine and other e-cigarette constituents. In 2020, several US states implemented comprehensive e-cigarette flavor bans to complement the federal e-cigarette flavor enforcement action that applied only to pod-based e-cigarette devices.

**Objective:**

To assess the association of state-level e-cigarette flavor bans with e-cigarette initiation across age groups in the US.

**Design, Setting, and Participants:**

This cross-sectional study conducted a difference-in-differences analysis accounting for variation in treatment timing using the Population Assessment of Tobacco and Health (PATH) Study survey waves 4 to 7 (January 2017 to April 2023). The PATH Study is an ongoing, nationally representative longitudinal cohort study of tobacco product use among noninstitutionalized US households. Adolescents (ages 12-17 years), young adults (ages 18-24 years), and adults (ages ≥25 years) who reported never using e-cigarettes at a baseline wave and completed the subsequent interview were included. Data were analyzed from January 2024 to June 2025.

**Exposures:**

A binary indicator for whether a state flavor ban was enacted as of the first day of the respondent baseline interview quarter.

**Main Outcomes and Measures:**

E-cigarette initiation was defined using the PATH Study variable *never-to-ever electronic nicotine product user*.

**Results:**

The study included 72 170 respondents (mean [SD] age, 45 [21] years; 36 893 female [54.4%; 95% CI, 53.7% to 55.5%]; 18 074 Hispanic [16.2%; 95% CI, 15.7% to 16.6%], 10 816 non-Hispanic Black [11.4%; 95% CI, 11.0% to 11.7%], and 34 749 non-Hispanic White [61.5%; 95% CI, 60.9% to 62.2%]). Among young adults, e-cigarette flavor bans were associated with a 6.05–percentage point (95% CI, −11.21 to −0.90 percentage point) decrease in initiation, representing a more than 50% decrease from the preban rate of 70 of 698 young adults (10.9%; 95% CI, 8.3% to 14.1%). However, no significant change was observed among adolescents or adults. Subgroup analyses of the young adult population revealed policy outcomes, as shown by adjusted difference-in-differences estimates, among populations typically considered to experience greater societal advantage, including non-Hispanic White individuals (−7.00 percentage points; 95% CI, −13.81 to −1.19 percentage points), young adults with higher household incomes (annual household income ≥$50 000; −6.92 percentage points; 95% CI, −13.12 to −0.73 percentage points), those without psychosocial distress (no internalizing symptoms: −5.58 percentage points; 95% CI, −11.00 to −0.16 percentage points; no externalizing symptoms: −7.07 percentage points; 95% CI, −12.01 to −2.13 percentage points), and individuals who were not members of sexual minority groups (−6.78 percentage points; 95% CI, −10.68 to −2.87 percentage points).

**Conclusions and Relevance:**

In this study, young adults living in states with e-cigarette flavor bans experienced significantly greater declines in e-cigarette initiation compared with those living in control states, but policies were not associated with change among adolescents or young adult subpopulations experiencing some form of societal disadvantage. These findings suggest that additional public health strategies are necessary to reduce adolescent e-cigarette initiation and ensure that current policies equitably benefit all populations.

## Key Points

**Question:**

Are state-level e-cigarette flavor bans associated with decreased e-cigarette initiation across age groups in the US?

**Findings:**

In this cross-sectional study among 72 170 adolescents, young adults, and adults who reported never using e-cigarettes, flavor bans were associated with a decrease in e-cigarette initiation among young adults compared with preban levels. No associations were observed among adolescents or adults aged 25 years and older.

**Meaning:**

In this study, state flavor bans were associated with decreased e-cigarette initiation among young adults but not among adolescents or more disadvantaged subgroups, suggesting a need for complementary public health strategies.

## Teaser

This cross-sectional study investigates the association of state-level flavored e-cigarette bans with e-cigarette initiation rates among adolescents, young adults, and adults.

## Introduction

E-cigarette use among young people has increased dramatically since the products were introduced to the US marketplace nearly 2 decades ago.^1^ At peak prevalence in 2019, 27.5% of US high school students reported current e-cigarette use,^2^ which poses a serious public health threat due to nicotine’s harmful effects on brain development and the potential to foster lifelong addiction.^3^ In 2021, 18.6% of young adults aged 18 to 24 years reported current e-cigarette use, substantially higher than the overall adult prevalence of 6.9%.^4^ Nearly 90% of adolescent and young adult e-cigarette users prefer flavored e-liquids (eg, fruit and mint), and flavors are frequently cited as a primary reason for initiation.^5,6^ Flavors enhance the palatability of e-cigarettes, making them more appealing to inexperienced users and lowering the threshold for experimentation.^7,8^ Thus, the availability of flavored products significantly contributed to the rise of e-cigarette use among young people.

In response to high rates of youth vaping, federal and state policymakers implemented regulations targeting flavored e-cigarettes. In January 2020, the US Food and Drug Administration issued a federal enforcement policy prohibiting the sale of flavored e-liquids in prefilled, single-use cartridges, primarily affecting pod-based devices.^9^ However, the policy had 2 major exceptions: menthol-flavored e-liquids remained available for pod-based devices, and the regulation did not apply to refillable systems or disposable devices. In parallel, several states adopted broader policy measures, enacting comprehensive bans on the sale of all flavored e-cigarettes regardless of device type and including menthol.^10^ Early evidence suggests that flavor-focused policies can be effective. Studies have found substantial reductions in flavored e-cigarette sales after implementation of such bans,^11,12,13,14,15^ indicating decreased product availability. Nonetheless, key questions remain about their association with behaviors in the general population. Young people may still obtain flavored products through social sources or online retailers or by traveling to areas without restrictions.^16^

Critically, there is a lack of evidence on whether these policies actually prevent e-cigarette initiation at the individual level. Most prior evaluations have relied on cross-sectional prevalence data before and after policy changes,^17,18,19,20^ which cannot determine whether fewer never users start using e-cigarettes. This study leveraged state-level flavor bans as natural experiments to assess their association with e-cigarette initiation among youths and young adults. Focusing on initiation, the transition from never to ever use, is essential for evaluating the effectiveness of flavor policies. While prevalence can fluctuate due to several factors, including cessation and product switching among current users, initiation directly captures new uptake, a primary target of prevention-focused policies. An effective flavor ban should be associated with a reduced likelihood that nonusers begin using e-cigarettes, even if overall prevalence changes more slowly.

In this context, this study examined whether state-level flavored e-cigarette sales bans were associated with decreased initiation across different age groups. Using longitudinal data from the Population Assessment of Tobacco and Health (PATH) Study (2017-2023, a period spanning the implementation of several state flavor bans), we assessed whether individuals living in states with bans were less likely to initiate e-cigarette use compared with peers in states without bans. We hypothesized that flavored e-cigarette bans would be associated with lower initiation rates. By focusing on individual-level transitions from never use to ever use, rather than population-level prevalence, this study offers a more direct test of whether flavor bans were associated with decreased initiation.

## Methods

This cross-sectional study was exempted for review and consent by the Institutional Review Board at the Wake Forest University School of Medicine because the study met the requirements of the 2019 Common Rule. We followed the Strengthening the Reporting of Observational Studies in Epidemiology (STROBE) reporting guideline.

### Data Sources

The primary data source was the PATH Study, an ongoing, nationally representative, longitudinal study of tobacco product use in US households.^21^ The longitudinal design allows researchers to track individual behavior changes across consecutive waves. The PATH Study includes adolescents (ages 12-17 years) and adults (ages 18-90 years), with oversampling of tobacco users, young adults (ages 18-24 years), and African American individuals.^22,23^ We used the restricted use file (RUF), which includes state identifiers, allowing us to determine whether participants lived in states with flavored e-cigarette bans. Details on study design, including consenting and assenting procedures, sampling strategies, weighting, and response rates, are available in the *PATH Study RUF User Guide*^24^ or previous publicaitons.^22^ We analyzed data from waves 4 (December 2016 to January 2018) through 7 (January 2022 to April 2023).

### Study Sample

To examine e-cigarette initiation, we limited the study sample to participants with 2 consecutive waves of data. We identified individuals who reported never using e-cigarettes at baseline waves (waves 4-6). Using the PATH Study ever use variable (R04M_EVR_ECIG for wave 4), we included only participants who had never used e-cigarettes according to the last wave they completed. We used corresponding markers to identify study samples for later waves. The study population comprised adolescents (ages 12-17 years), young adults (ages 18-24 years), and adults (ages ≥25 years) who reported no prior e-cigarette use at baseline. We excluded states with fewer than 100 never e-cigarette users during the study period to avoid unreliable estimates from small sample sizes. As a result, 342 of 122 779 respondents (0.3%) from 11 states were excluded, including 1 state with a ban. The final sample included 4 states that implemented flavored e-cigarette sales bans (treatment states) and 36 states without bans (control states). For additional details, see the eMethods in Supplement 1.

### Outcome

For e-cigarette–naive participants identified at baseline, we assessed e-cigarette initiation by examining self-reported electronic nicotine product use at subsequent wave interviews (waves 4.5-7), consistent with prior research.^25^ The outcome variable was a binary indicator of e-cigarette initiation, defined as transitioning from never to ever electronic nicotine product use at follow-up (R05R_A_NEW_EPRODS for the adult sample identified at wave 4). Variables used to define outcomes for each follow-up wave are described in the eMethods in Supplement 1.

### Exposure

The exposure variable was a binary indicator of whether respondents were subject to flavored e-cigarette bans at the time of their baseline interview. Five states implemented state-level bans on flavored e-cigarette sales during the study period (January 2017 to December 2021): Maryland, Massachusetts, New Jersey, New York, and Rhode Island (excluded due to small sample size). Flavored e-cigarette bans took effect between quarter 3 2019 and quarter 2 2020.^26^ We used state identifiers to categorize participants residing in these 4 states. Exposure was coded as 1 if participants resided in 1 of the 4 states and a flavor ban was enacted as of the first day of the quarter in which their baseline interview occurred. Exposure was coded as 0 for participants residing in states without flavor bans and for participants residing in states with flavor bans but who were interviewed before the policies went into effect.

### Covariates

Using the socioecological model as a guide,^27^ we identified 4 covariate domains of behavioral influence: sociodemographic and psychosocial distress (intrapersonal level) and state tobacco-control policies, economic climate, and public health emergency events (societal level). Sociodemographic variables included age, sex assigned at birth, race and ethnicity, household income, parental education (youth sample) and educational attainment (young adult and adult samples), and sexual minority status (lesbian, gay, and bisexual [LGB] vs non-LGB).^28^ Race and ethnicity were self-reported. Participants reported race (Asian alone, Black alone, White alone, or other race, including multiracial) and ethnicity (Hispanic or non-Hispanic). These were combined to create 4 categories: Hispanic, non-Hispanic Black, non-Hispanic White, and other (Asian, other races, or multiracial). Asian, other races, and multiracial were combined due to low population numbers. Race and ethnicity were assessed to examine potential differences in e-cigarette initiation and policy outcomes across demographic groups. Psychosocial distress was assessed using 4 internalizing symptoms (depression, sleep disturbances, anxiety, and emotional distress) and 7 externalizing symptoms (lying and conning, attention difficulties, listening issues, bullying, fights, restlessness, and impulsivity) measured by the Global Appraisal of Individual Needs–Short Screener,^29^ a validated mental health–screening tool.^30^ We categorized psychosocial distress into 3 groups: no symptoms, 1 to 2 symptoms, and 3 or more symptoms. Tobacco-control policies included binary variables for 2 measures: minimum age requirements of 21 years for purchasing tobacco products (T21 laws) and e-cigarette taxes in a state at a given time. We considered state and federal T21 laws. Data on T21 laws and e-cigarette taxes were from the Preventing Tobacco Addiction Foundation and published articles, respectively.^31,32^ We also measured state tobacco-control funding as a percentage of Centers for Disease Control and Prevention (CDC)–recommended funding levels using data from the Campaign for Tobacco-Free Kids.^33,34^ Public health emergency events included the e-cigarette, or vaping product, use–associated lung injury (EVALI) outbreak and COVID-19 pandemic, which may have differentially affected states. State economic climate includes poverty and unemployment rates. For each state, we adjusted for EVALI hospitalizations (June 2019 to February 2020) using CDC data^35^ and cumulative COVID-19 deaths per 100 000 population from quarter 1 2020 onward using the CDC COVID Data Tracker^36^ and state pandemic closure.^37^ Additional details are available in the eMethods in Supplement 1.

### Statistical Analysis

In this repeated cross-sectional study, we used the Gardner 2-stage difference-in-differences (DiD) approach^38^ to compare e-cigarette initiation rates in states with and without flavored e-cigarette sales bans before (2017-2019) and after (2020-2021) policy implementation. This method accounts for potential bias in standard DiD models when treatment effect sizes vary across groups and over time. Models adjusted for state, year, and quarter fixed effects in addition to aforementioned covariates of sociodemographic, psychosocial distress, state tobacco-control policies, and public health emergency events. Because logistic regressions with large numbers of fixed effects can introduce bias,^39^ we specified linear probability models instead. We applied weights to adjust for the complex sampling design^40^ and estimated standard errors clustered at the state level, robust to heteroscedasticity using the Huber-White variance estimator.^41,42^ To examine policy outcomes, we conducted DiD analyses for each age group (12-17, 18-24, and ≥25 years) separately (eMethods in Supplement 1). To assess heterogeneity in policy outcomes, we estimated models including interaction of the exposure variable with sex, race and ethnicity, household income, psychosocial distress, and sexual minority status. We assessed the parallel trend assumption using event study specifications. Finally, we conducted several sensitivity analyses (eMethods in Supplement 1) to examine the robustness of our findings. We used Stata statistical software version 18 (StataCorp) to conduct analyses. Statistical tests were 2-tailed, and *P* < .05 was considered statistically significant. Data were analyzed from January 2024 to June 2025.

## Results

### Sample Characteristics

The study included 72 170 PATH respondents (mean [SD] age, 45 [21] years; 36 893 female [54.4%; 95% CI, 53.7%-55.5%]; 18 074 Hispanic [16.2%; 95% CI, 15.7%-16.6%], 10 816 non-Hispanic Black [11.4%; 95% CI, 11.0%-11.7%], and 34 749 non-Hispanic White [61.5%; 95% CI, 60.9%-62.2%]) who reported never using e-cigarettes at baseline interviews conducted between 2017 and 2021. The sample consisted of 34 893 youths (48.3%), 13 369 young adults (18.5%), and 23 908 adults (33.1%). Table 1 compares participant characteristics by state flavored e-cigarette ban status, showing raw counts and weighted percentages. More than half of participants reported annual household incomes of $50 000 or more (36 421 participants [55.0%; 95% CI are (54.3%-55.7%]), and 6028 participants (5.5%; 95% CI, 5.2%-5.7%) self-identified as members of sexual minority groups. Several demographic differences emerged between states with and without flavored e-cigarette bans: states with bans had higher proportions of female participants, non-Hispanic Black respondents, and individuals with annual household incomes of more than $100 000. Approximately one-third of respondents reported past-month psychosocial distress (internalizing psychosocial distress: 30 368 participants [34.3%; 95% CI, 33.7%-34.9%]; externalizing psychosocial distress: 34 247 participants [38.0%; 95% CI, 37.4%-38.6%]), with no significant differences by state policy. However, states with flavor bans had lower tobacco-control spending (<25% of CDC-recommended levels) and reported higher numbers of EVALI cases than states without flavor bans.

**Table 1.** Study Sample Baseline Characteristics^a^

Characteristic	Participants, No. (% [95% CI])			*P* value
	Total (N = 72 170)	Treatment (n = 6538)^b^	Control (n = 65 632)^b^	
Interview year				
2017	22 363 (30.7 [30.3-31.5])	2052 (31.5 [29.6-33.5])	20 311 (30.9 [30.2-31.5])	.23
2018	12 617 (10.3 [10.1-10.6])	1108 (11.1 [10.3-12])	11 509 (10.3 [10-10.7])	
2019	14 589 (20.3 [20.8-21.8])	1323 (20.4 [18.8-22.1])	13 266 (20.4 [20.9-2.2])	
2020	6716(3.8 [3.7-3.9])	607 (3.9 [3.6-4.3])	6109 (3.8 [3.7-3.9])	
2021	15 885 (33.5 [32.8-34.2])	1448 (32.6 [30.5-34.8])	14 437 (33.6 [32.9-34.3])	
Age, mean (SD), y	45.074 (21.215)	45.277 (21.195)	45.050 (21.217)	.61
Sex				
Male	35 277 (45.6 [45-46.3])	3013 (43.5 [41.4-45.7])	32 264 (45.9 [45.2-46.6])	.04
Female	36 893 (54.4 [53.7-55])	3525 (56.5 [54.3-58.6])	33 368 (54.1 [53.4-54.8])	
Race and ethnicity^c^				
Hispanic	18 074 (16.2 [15.7-16.6])	1538 (15.8 [14.3-17.4])	16 536 (16.2 [15.8-16.7])	<.001
Non-Hispanic Black	10 816 (11.4 [11-11.7])	1212 (16.3 [15.1-17.7])	9604 (10.8 [10.4-11.1])	
Non-Hispanic White	34 749 (61.5 [60.9-62.2])	2819 (54 [51.9-56.1])	31 930 (62.5 [61.8-63.1])	
Other racial identity	8531(10.9 [10.5-11.4])	969 (13.9 [12.4-15.6])	7562 (10.5 [10.1-11])	
Annual household income, $1000				
<25	17 152 (20 [19.5-20.6])	1268 (18.2 [16.5-20.0])	15 884 (20.3 [19.7-20.8])	<.001
25-50	14 669 (19.5 [19-20])	1100 (16.8 [15.3-18.5])	13 569 (19.8 [19.3-20.4])	
50-100	17 448 (27.1 [26.5-27.7])	1400 (23.4 [21.4-25.0])	16 048 (27.5 [26.9-28.2])	
≥100	18 973 (27.9 [27.4-28.5])	2300 (33.7 [31.7-35.7])	16 673 (27.3 [26.6-27.9])	
Other or unknown	3928 (5.5 [5.2-5.8])	470 (8.2 [7.1-9.6])	3458 (5.1 [4.8-5.5])	
Educational attainment				
Without high-school diploma or with GED	5196 (9.9 [9.5-10.3])	466 (10.5 [9.1-12.1])	4730 (9.8 [9.4-10.3])	<.001
High-school diploma	9096 (17.8 [17.2-18.4])	757 (16.4 [14.7-18.3])	8339 (18.0 [17.4-18.6])	
Some college degree	12 165 (24.5 [24-25.1])	1002 (20.5 [18.8-22.3])	11 163 (25.0 [24.4-25.7])	
Bachelor’s degree	10 820 (31.5 [30.8-32.1])	1307 (36.7 [34.6-38.8])	9513 (30.8 [30.2-31.5])	
Not applicable^d^	34 893 (16.2 [16-16.5])	3006 (15.8 [14.9-16.7])	31 887 (16.3 [16.0-16.6])	
Sexual minority group				
No	56 141 (89.6 [89.3-89.9])	5079 (89.5 [88.5-90.4])	51 062 (89.6 [89.3-89.9])	.28
Yes	6028(5.5 [5.2-5.7])	604 (5.9 [5.1-6.8])	5424 (5.4 [5.2-5.7])	
Missing	286 (0.1 [0.1-0.2])	24 (0.1 [0.1-0.2])	262 (0.1 [0.1-0.2])	
Not applicable^e^	9715 (4.8 [4.7-4.9])	831 (4.5 [4.2-4.9])	8884 (4.8 [4.7-4.9])	
Presence of state or federal Tobacco 21 law^f^				
No	41 454 (53.2 [52.6-53.9])	3842 (54.2 [52-56.3])	37 612 (53.1 [52.4-53.8])	.36
Yes	30 716 (46.8 [46.1-47.4])	2696 (45.8 [43.7-48])	28 020 (46.9 [46.2-47.6])	
Presence of e-cigarette tax				
No	48 275 (63.7 [63.1-64.4])	4350 (59.2 [57-61.4])	43 925 (64.3 [63.6-64.9])	<.001
Yes	23 895 (36.3 [35.6-36.9])	2188 (40.8 [38.6-43])	21 707 (35.7 [35.1-36.4])	
Tobacco-control spending level, % of CDC recommendation				
≤25	52 014 (73.3 [72.7-73.9])	6538 (100)	45 476 (70.1 [69.4-70.7])	<.001
26-50	12 960 (17.3 [16.8-17.8])	0	12 960 (19.4 [18.9-19.9])	
51-75	4273 (7.0 [6.6-7.4])	0	4273 (7.8 [7.4-8.3])	
>75	2923 (2.4 [2.3-2.6])	0	2923 (2.7 [2.5-2.3])	
No. of internalizing psychosocial distress symptoms experienced in the past mo^g^				
0	41 802 (65.7 [65.1-66.3])	3849 (67.1 [65.1-69.0])	37 953 (65.5 [64.9-66.2])	.13
1-2	19 722 (25 [24.4-25.6])	1794 (24.6 [22.9-26.4])	17 928 (25.0 [24.4-25.6])	
≥3	10 646 (9.3 [9.0-9.6])	895 (8.3 [7.4-9.4])	9751 (9.4 [9.1-9.8])	
No. of externalizing psychosocial distress symptoms experienced in the past mo^g^				
0	37 923 (62 [61.4-62.6])	3527 (63.1 [61.1-65.1])	34 396 (61.9 [61.2-62.5])	.46
1-2	22 392 (28.2 [27.6-28.7])	1968 (27.3 [25.5-29.2])	20 424 (28.3 [27.6-28.9])	
≥3	11 855 (9.8 [9.5-10.2])	1043 (9.5 [8.6-10.6])	10 812 (9.9 [9.5-10.2])	
No. of hospitalized EVALI cases reported to CDC as of February 18, 2020, mean (SD)	2.415 (1.929)	2.765 (2.154)	2.373 (1.896)	<.001
Log of cumulative COVID-19 deaths, mean (SD)	3.585 (4.692)	3.556 (4.693)	3.588 (4.691)	.77

Abbreviations: CDC, Centers for Disease Control and Prevention; EVALI, e-cigarette, or vaping product, use–associated lung injury; GED, General Educational Development test.

^a^ Raw counts and weighted percentages were reported.

^b^ Treatment states include MA, MD, NJ, and NY. Control states include AL, AR, AZ, CA, CO, CT, FL, GA, HI, IA, IL, IN, KY, LA, ME, MI, MN, MO, MS, MT, NC, NE, NH, NV, OH, OK, OR, PA, SC, TN, TX, UT, VA, WA, WI, and WV. CA was a control state in this analyses because its state-level ban was enacted in December 2022. Treatment status was coded as 1 if participants lived in 1 of the 4 treatment states on the first day of the quarter when their baseline interview occurred. It was coded as 0 (control) for participants in control states or in treatment states who were interviewed before the policy took effect.

^c^ Participants reported race (White alone, Black alone, Asian alone, or other race, including multiracial) and ethnicity (Hispanic or non-Hispanic). These were combined to create 4 categories: Hispanic, non-Hispanic Black, non-Hispanic White, and other (Asian, other races, or multiracial).

^d^ Educational attainment was not applicable for youth participants.

^e^ Sexual orientation questions were asked only of youths aged 14 years or older, so sexual minority status was not applicable for those aged 12 to 13 years.

^f^ State legislation that prohibited retailers from selling tobacco products, including e-cigarettes and vaping products, to anyone younger than 21 years and federal legislation enacted in December 2019.

^g^ Psychosocial distress was measured by the Global Appraisal of Individual Needs–Short Screener. The survey assesses 4 internalizing symptoms (depression, sleep disturbances, anxiety, and emotional distress) and 7 externalizing symptoms (lying and conning, attention difficulties, listening issues, bullying, fights, restlessness, and impulsiveness).

### Main Analyses

The Figure depicts quarterly e-cigarette initiation among young adults in treatment and control states from 2017 to 2021. The vertical line at 2019 quarter 4 indicates the earliest implementation of state-level flavored e-cigarette sales bans in treatment states. No data were available for young adults in the first three 2018 quarters because wave 4.5 included only youths. No data were collected during the first two 2020 quarters due to the COVID-19 pandemic. Prior to the bans, initiation rates were generally higher in treatment states but followed a similar, albeit noisy, downward trend in both groups, declining from 23 of 123 participants (18.6%; 95% CI, 10.6% to 24.5%) in the treatment group and 137 of 1255 participants (10.9%; 95% CI, 9.0% to 12.8%) in the control group in early 2017 to 3 of 46 participants (6.4%; 95% CI, 0.1% to 19.2%) and 4 of 89 participants (4.3%; 95% CI, <0.1% to 9.6%), respectively, by late 2019. (Numerators and denominators are raw numbers, and percentages are weighted.) After policy implementation, e-cigarette initiation in treatment states continued to decline, reaching approximately 2% by 2021 quarter 4 (2 of 105 participants [2.1%; 95% CI, <0.1% to 6.7%]), while rates in control states rebounded and remained consistently higher than those in treatment states throughout the rest of the study period.

**Figure.**
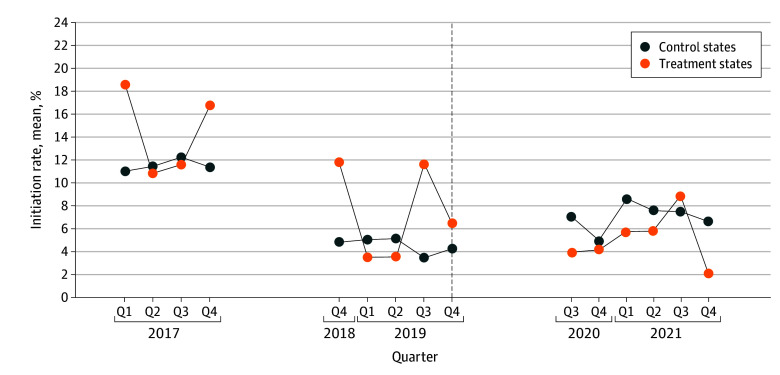
Mean State-Level E-Cigarette Initiation Rates Among Young Adults The line graph depicts e-cigarette initiation rates for young adults (ages 18-24 years) from quarter 1 (Q1) 2017 to Q4 2021 comparing states with and without flavor bans. Treatment states include MA, MD, NJ, and NY. Control states include AL, AR, AZ, CA, CO, CT, FL, GA, HI, IA, IL, IN, KY, LA, ME, MI, MN, MO, MS, MT, NC, NE, NH, NV, OH, OK, OR, PA, SC, TN, TX, UT, VA, WA, WI, and WV. CA was a control state in this analyses because its state-level ban was enacted in December 2022. Data for young adults were unavailable for the first three 2018 quarters because wave 4.5 included only youths. No data were collected during the first two 2020 quarters due to the COVID-19 pandemic.

Table 2 presents DiD estimates of the association between state-level flavored e-cigarette sales bans and e-cigarette initiation across 3 age groups. Among young adults, bans were associated with a 6.05–percentage point (95% CI, −11.21 to −0.90 percentage points) decrease in initiation, representing a decrease of more than 50% from the preban rate of 70 of 698 young adults (10.9%; 95% CI, 8.3%-14.1%) in treatment states. However, no significant change was observed among adolescents or adults aged 25 years or older. Event-study results confirmed that states with and without bans followed parallel trends before the policy took effect (eFigure and eTable 1 in Supplement 1).

**Table 2.** Association of State-Level Flavored E-Cigarette Sales Bans With E-Cigarette Initiation

Age group	Treatment states (n = 4)^a^			Control states (n = 36)			Differential change			
	Initiation rate, No. (% [95% CI])^b^		Change, pp	Initiation rate, No. (% [95% CI])^b^		Change, pp	Unadjusted DiD, pp	*P* value	Adjusted DiD, pp (95% CI)	*P* value
	Before ban^c^	After ban		Before ban^c^	After ban					
Youths (12 to 17 y)	2208 (8.9 [7.7-10.2])	798 (6.3 [4.7-8.4])	−2.60	23 742 (9.5 [9.1-9.9])	8145 (6.4 [5.9-7.1])	−3.01	0.41	.73	−0.56 (−1.70 to 2.83)	.63
Young adults (18 to 24 y)	698 (10.9 [8.3-14.1])	539 (5.7 [4-8.1])	−5.12	6495 (8.6 [7.8-9.3])	5637 (7.2 [6.4-7.9])	−1.40	−3.72	.05	−6.05 (−11.21 to −0.90)	.02
Adults (≥25 y)	1544 (0.7 [0.4-1.0])	751 (1 [0.4-2.3])	0.33	14 346 (1.3 [1.1-1.5])	7267 (1 [0.8-1.2])	−0.36	0.69	.13	1.04 (−0.226 to 2.34)	.12

Abbreviations: DiD, difference-in-differences; pp, percentage points.

^a^ Treatment states include MA, MD, NJ, and NY. Control states include AL, AR, AZ, CA, CO, CT, FL, GA, HI, IA, IL, IN, KY, LA, ME, MI, MN, MO, MS, MT, NC, NE, NH, NV, OH, OK, OR, PA, SC, TN, TX, UT, VA, WA, WI, and WV. CA was a control state in this analyses because its state-level ban was enacted in December 2022, after the current study period.

^b^ Numbers represent the number of never e-cigarette users identified at baseline interviews (unweighted counts). Numbers in parentheses represent weighted percentages.

^c^ The preban period spanned from 2017 quarter 1 to 2019 quarter 3 for Massachusetts and New York, from 2017 quarter 1 to 2020 quarter 1 for Maryland, and from 2017 quarter 1 to 2020 quarter 2 for New Jersey. The postban period extended from 2019 quarter 4 to 2021 quarter 4 for Massachusetts and New York, from 2020 quarter 2 to 2021 quarter 4 for Maryland, and from 2020 quarter 3 to 2021 quarter 4 for New Jersey.

We also conducted several sensitivity analyses to assess the robustness of our findings. First, we excluded 1 treatment state at a time to ensure that results were not driven by any single state. Second, we extended the study period to include 2016 to capture earlier trends in flavored e-cigarette use. Third, we excluded Washington and Montana, which implemented temporary emergency bans on flavored e-cigarette sales in response to the 2019 EVALI crisis,^43,44^ and Utah, which restricted flavored e-cigarette sales to tobacco specialty retailers.^26^ Fourth, we excluded bordering states to assess potential bias from cross-state purchasing. Fifth, we excluded California and Minnesota to examine outcomes associated with local e-cigarette flavor bans in control states. Sixth, we stratified the young adult age group into individuals aged 18 to 20 years and 21 to 24 years to examine the federal T21 law. Results remained consistent across all sensitivity analyses (eTable 2 in Supplement 1).

### Subgroup Analyses

To explore potential heterogeneity in policy outcomes, we conducted subgroup analyses among young adults by sex assigned at birth, self-reported race and ethnicity, household income, psychosocial status, and sexual minority status. As shown in Table 3, although policy outcomes did not appear to vary by sex, decreases in e-cigarette initiation were concentrated in certain subgroups. Significant decreases were observed among non-Hispanic White young adults (−7.00 percentage points; 95% CI, −13.81 to −0.19 percentage points) and other racial and ethnic groups (−9.40 percentage points; 95% CI, −14.31 to −4.48 percentage points), among whom Asian participants made up the majority (4412 of 8531 participants with other race were Asian [51.7%]), while changes among Black and Hispanic subgroups were not statistically significant. Young adults with annual household incomes greater that $50 000 experienced a 6.92–percentage point (95% CI, −13.12 to −0.73 percentage points) decrease, with no associations observed in the lower-income group. The policy association appeared concentrated among individuals without externalizing symptoms (−7.07 percentage points; 95% CI, −12.01 to −2.13 percentage points), while associations were observed in groups with (−6.78 percentage points; 95% CI, −11.72 to −1.83 percentage points) and without (−5.58 percentage points; 95% CI, −11.00 to −0.16 percentage points) internalizing symptoms. Non-LGB individuals showed a significant 6.78–percentage point (95% CI, −10.68 to −2.87 percentage points) decrease in initiation, whereas no association was detected among LGB participants. Subgroup analyses among youths revealed no significant policy outcomes across nearly all examined groups, consistent with the main findings (eTable 3 in Supplement 1). A sensitivity analysis among participants aged 21 to 24 years found a decrease in e-cigarette initiation similar to the main findings (eTable 4 in Supplement 1).

**Table 3.** Association of State-Level Flavored E-Cigarette Sales Bans With E-Cigarette Initiation Among Young Adults (18-24)

Young adult characteristic	Treatment states (n = 4)^a^			Control states (n = 36)			Differential change			
	Initiation rate No. (% [95% CI])^b^		Change, pp	Initiation rate No. (% [95% CI])^b^		Change, pp	Unadjusted DiD, pp	*P* value	Adjusted DiD, pp (95% CI)	*P* value
	Before ban^c^	After ban		Before ban^c^	After ban					
Overall	698 (10.9 [8.3 to 14.1])	539 (5.7 [4.0 to 8.1])	−5.12	6495 (8.6 [7.8 to 9.3])	5637 (7.2 [6.4 to 7.9])	−1.40	−3.72	.05	−6.05 (−11.21 to −0.90)	.02
Sex										
Female	397 (8.3 [5.1 to 11.5])	288 (5.3 [2.8 to 7.8])	−3.08	3592 (7.7 [6.8 to 8.7])	2897 (7.9 [6.8 to 9.0])	0.16	−3.24	.14	−6.31 (−12.54 to −0.08)	.05
Male	301 (14.1 [9.0 to 19.3])	251 (6.3 [3.1 to 9.6])	−7.79	2903 (9.6 [8.4 to 10.7])	2740 (6.4 [5.4 to 7.5])	−3.15	−4.64	.15	−5.74 (−10.26 to −1.22)	.01
Race and ethnicity										
Hispanic	182 (14.3 [8.6 to 19.9])	150 (7.4 [3.4 to 11.4])	−1.67	1953 (8.9 [7.8 to 10.0])	1819 (7.6 [6.4 to 8.8])	−2.37	0.70	.85	−3.14 (−9.43 to 3.14)	.33
Non-Hispanic Black	177 (5.6 [1.6 to 9.5])	116 (3.8 [0.8 to 6.8])	−1.78	1195 (8.1 [6.4 to 9.9])	975 (7.4 [5.5 to 9.2])	−0.78	−1.00	.73	−5.40 (−11.03 to 0.23)	.06
Non-Hispanic White	230 (9.9 [4.9 to 15.0])	180 (8.3 [3.6 to 12.9])	−6.89	2679 (8.9 [7.5 to 10.3])	2197 (6.5 [5.3 to 7.7])	−1.26	−5.63	.12	−7.00 (−13.81 to −0.19)	.04
Other racial identity	109 (10.5 [4.3 to 16.7])	93 (2.0 [−0.6 to 4.6])	−8.46	668 (7.1 [5.0 to 9.2])	646 (6.5 [4.4 to 8.5])	−0.64	−7.82	.04	−9.40 (−14.31 to −4.48)	<.001
Annual household income, $1000										
<50	372 (10.0 [6.0 to 14.08])	183 (6.1 [2.6 to 9.5])	−3.99	3824 (7.9 [7.0 to 8.8])	2699 (7.1 [6.0 to 8.1])	−0.83	−3.16	.26	−4.75 (−11.00 to 1.50)	.14
≥50	261 (13.2 [8.4 to 18.1])	254 (5.6 [2.8 to 8.4])	−7.66	2039 (9.2 [7.9 to 10.5])	2161 (7.7 [6.4 to 9.0])	−1.54	−6.12	.04	−6.92 (−13.12 to −0.73)	.03
Unknown	65 (4.5 [<0.0 to 9.1])	102 (5.6 [0.4 to 10.7])	1.04	632 (10.3 [7.5 to 13.1])	777 (5.99 [4.3 to 7.6])	−4.30	5.34	.17	−6.35 (−10.87 to −1.83)	.006
Psychosocial destress^d^										
Any internalizing symptom	269 (11.0 [6.2 to 15.73])	233 (6.3 [3.1 to 9.5])	−4.68	2696 (10.0 [8.7 to 11.3])	2352 (8.3 [7.1 to 9.4])	−1.76	−2.92	.34	−6.78 (−11.72 to −1.83)	.007
No internalizing symptom	429 (10.8 [7.16 to 14.4])	32 (5.3 [2.8 to 7.9])	−5.46	3799 (7.5 [6.7 to 8.4])	3285 (6.4 [5.4 to 7.3])	−1.18	−4.28	.07	−5.58 (−11.00 to −0.16)	.04
Any externalizing symptom	299 (12.0 [7.2 to 16.8])	247 (7.8 [4.4 to 11.2])	−4.22	2988 (9.6 [8.5 to 10.8])	2762 (8.5 [7.3 to 9.6])	−1.15	−3.07	.32	−4.98 (−11.07 to 1.12)	.11
No externalizing symptom	399 (9.9 [6.4 to 13.4])	292 (3.9 [1.6 to 6.2])	−5.98	3507 (7.6 [6.7 to 8.6])	2875 (5.9 [4.9 to 6.8])	−1.78	−4.20	.06	−7.07 (−12.01 to −2.13)	.005
Sexual minority status										
LGB	99 (8.5 [2.4 to 14.5])	96 (10.4 [4.2 to 16.6])	1.93	767 (10.0 [7.7 to 12.3])	867 (8.3 [6.3 to 10.3])	−1.72	3.65	.44	−2.51 (−16.52 to 11.50)	.73
Non-LGB	599 (11.2 [8.0 to 14.4])	442 (4.8 [2.7 to 6.8])	−6.40	5728 (8.4 [7.6 to 9.2])	4770 (7.0 [6.1 to 7.8])	−1.43	−4.97	.01	−6.78 (−10.68 to −2.87)	.001

Abbreviations: LGB, lesbian, gay, and bisexual; pp, percentage points.

^a^ Treatment states include MA, MD, NJ, and NY. Control states include AL, AR, AZ, CA, CO, CT, FL, GA, HI, IA, IL, IN, KY, LA, ME, MI, MN, MO, MS, MT, NC, NE, NH, NV, OH, OK, OR, PA, SC, TN, TX, UT, VA, WA, WI, and WV. CA was a control state in this analyses because its state-level ban was enacted in December 2022.

^b^ Numbers represent the number of never e-cigarette users identified at baseline interviews (unweighted counts). Numbers in parentheses represent weighted percentages.

^c^ The preban period spanned from 2017 quarter 1 to 2019 Q3 for Massachusetts and New York, from 2017 quarter 1 to 2020 quarter 1 for Maryland, and from 2017 quarter 1 to 2020 quarter 2 for New Jersey. The postban period extended from 2019 quarter 4 to 2021 quarter 4 for Massachusetts and New York, from 2020 quarter 2 to 2021 quarter 4 for Maryland, and from 2020 quarter 3 to 2021 quarter 4 for New Jersey.

^d^ Psychosocial distress was measured by the Global Appraisal of Individual Needs–Short Screener. The survey assesses 4 internalizing symptoms (depression, sleep disturbances, anxiety, and emotional distress) and 7 externalizing symptoms (lying and conning, attention difficulties, listening issues, bullying, fights, restlessness, and impulsiveness).

## Discussion

In this cross-sectional study examining the association between state-level flavored e-cigarette sales bans and e-cigarette initiation across age groups, 2 key themes emerged. First, results partially support the hypothesis that such bans are associated with decreased e-cigarette initiation. Young adults in states with flavor bans experienced significantly greater declines in initiation compared with young adults in control states, representing a decrease of more than 50% from preban levels. In contrast, no significant changes were observed among adolescents or older adults. Second, subgroup analyses indicated that policy outcomes were concentrated among subpopulations typically considered to experience greater societal advantage, including non-Hispanic White individuals, young adults with higher household incomes, those without psychosocial distress, and those who were not members of sexual minority groups, highlighting potential equity concerns for flavor policies.

Our findings align with a growing body of evidence showing that flavor restrictions were associated with decreased retail sales of banned products.^11,12,15,45,46,47^ Studies of statewide bans in Massachusetts, New York, and Rhode Island have documented 25% to 50% declines in flavored e-cigarette sales within months of ban implementation.^11^ Consistent with these market trends, we observed a substantial decline in e-cigarette initiation among young adults living in states with bans, reinforcing recent research associating flavor ban policies with lower prevalence in this age group.^17,18,19^ The absence of a policy outcome among adolescents also mirrors findings from multistate evaluations from 2024 to 2025^17,18,48^ reporting little to no change in youth e-cigarette use after flavor bans. Because youths are age restricted from purchasing tobacco products already via T21 laws, additional flavor bans may do little to further limit this group’s access, which is frequently via informal or illicit channels, thus making retail bans easier to circumvent.^49^ Indeed, a 2025 meta-analysis^50^ reported null policy outcomes for T21 laws in youth tobacco use. Our sensitivity analysis, limited to participants aged 21 to 24 years, further supported our main finding. Given that this age group was unaffected by the federal T21 law, which raised the minimum purchase age to 21 years, the observed decrease in e-cigarette initiation within this group suggests that e-cigarette flavor bans, rather than the T21 law, were the key factor associated with lower young adult initiation rates.

A notable and concerning finding is that changes associated with the policy were concentrated among young adults belonging to populations typically considered to experience greater societal advantage. Social determinants frameworks offer insight into this disparity. When flavored e-cigarettes are restricted, individuals from marginalized communities may be more likely to obtain products through informal networks, cross-border purchases, or illicit markets.^51^ Prior studies have shown that flavor bans were less effective in areas with high tobacco retailer density, which is common in lower-income and more racially diverse communities.^52,53^ Higher socioeconomic status is often associated with greater responsiveness to tobacco-control policies, including greater awareness, risk perceptions, and adherence.^54^ In the context of e-cigarettes, individuals with higher education and income levels may be more likely to reduce use after flavor bans due to reduced exposure and higher receptivity to regulation.^55^ Conversely, members of sexual minority groups face increased baseline rates of e-cigarette use and may not respond similarly to such policies. One study^56^ found that low perceived harm from occasional e-cigarette use was a strong predictor of use among students who were members of sexual minority groups, suggesting that access restrictions alone may be insufficient for this group. Similarly, individuals experiencing psychosocial distress may use nicotine products for self-regulation or coping, potentially making these individuals less responsive to product-based restrictions like flavor bans.^57,58^ These findings highlight the need for complementary, equity-focused interventions to address broader social and behavioral drivers of nicotine use.

### Limitations

Our study has several limitations. First, initiation was based on self-reported data, which may be subject to recall errors and social desirability bias. Second, although we adjusted for a broad set of covariates, unmeasured confounding remains possible and may bias estimated policy outcomes. Third, local flavor restrictions may have affected our analysis. However, sensitivity analyses excluding states with the most local flavor bans produced similar results. Fourth, our definition of initiation (never use to ever use) encompasses a range of use patterns from experimentation to daily use. Future studies should examine the association of e-cigarette flavor bans with frequency of use. Fifth, the lack of data from early 2020 due to the COVID-19 pandemic is another limitation. However, the Gardner DiD approach helps mitigate this by flexibly accounting for variation in treatment timing and missing periods.

## Conclusions

In this cross-sectional, study, state-level flavored e-cigarette bans were associated with decreased initiation among young adults. However, no associations were observed among adolescents or disadvantaged young adults. The study underscores the importance of closing regulatory loopholes to maximize public health outcomes associated with e-cigarette flavor policies.

## Notes

Supplement 1. eMethods. eFigure. Parallel Trend Assumption Test: Main Analyses Across 3 Age Groups and Subgroup Analyses Limited to Young Adults (18-24) eTable 1. Event Study Analysis of Statewide Flavored E-Cigarette Sales Bans and E-cigarette Initiation eTable 2. Sensitivity Analysis: Association of Statewide Flavored E-Cigarette Sales Bans With Vaping Initiation Among Young Adults (18-24) eTable 3. Subgroup Analysis: Association of Statewide Flavored E-Cigarette Sales Bans With Vaping Initiation Among Youths (12-17) eTable 4. Association of State-Level Flavored E-Cigarette Sales Bans With Initiation Among Young Adults (18-24), Stratified by Age (18-20 vs 21-24) eReferences.

Supplement 2.
Data Sharing Statement
