# Supplementary material for: State-Level Flavored E-Cigarette Bans and Initiation Rates Among Youths and Adults
Source: JAMA Netw Open. 2026 Jan 5;9(1):e2551744. doi: 10.1001/jamanetworkopen.2025.51744 (PMC12771250; doi:10.1001/jamanetworkopen.2025.51744)
Supplement: Supplement 1. — eMethods. eFigure. Parallel Trend Assumption Test: Main Analyses Across 3 Age Groups and Subgroup Analyses Limited to Young Adults (18-24) eTable 1. Event Study Analysis of Statewide Flavored E-Cigarette Sales Bans and E-cigarette Initiation eTable 2. Sensitivity Analysis: Association of Statewide Flavored E-Cigarette Sales Bans With Vaping Initiation Among Young Adults (18-24) eTable 3. Subgroup Analysis: Association of Statewide Flavored E-Cigarette Sales Bans With Vaping Initiation Among Youths (12-17) eTable 4. Association of State-Level Flavored E-Cigarette Sales Bans With Initiation Among Young Adults (18-24), Stratified by Age (18-20 vs 21-24) eReferences. [file jamanetwopen-e2551744-s001.pdf]

Supplemental Online Content

Lin M-Y, Abdelfattah LI, Hanchate AD, Sutfin EL, Denlinger-Apte RL. State-Level Flavored E-Cigarette Bans and Initiation Rates Among Youths and Adults. JAMA Netw Open. 2026;9(1):e2551744. doi:10.1001/jamanetworkopen.2025.51744

eMethods.

eFigure. Parallel Trend Assumption Test: Main Analyses Across 3 Age Groups and Subgroup Analyses Limited to Young Adults (18-24)

eTable 1. Event Study Analysis of Statewide Flavored E-Cigarette Sales Bans and E-cigarette Initiation

eTable 2. Sensitivity Analysis: Association of Statewide Flavored E-Cigarette Sales Bans With Vaping Initiation Among Young Adults (18-24)

eTable 3. Subgroup Analysis: Association of Statewide Flavored E-Cigarette Sales Bans With Vaping Initiation Among Youths (12-17)

eTable 4. Association of State- Level Flavored E-Cigarette Sales Bans With Initiation Among Young Adults (18-24), Stratified by Age (18-20 vs 21-24)

eReferences.

This supplemental material has been provided by the authors to give readers additional information about their work.

© 2026 Lin M-Y et al. *JAMA Network Open*.

eMethods

Control and treatment states

The study included four treatment states that implemented statewide bans on the sale of flavored e-cigarettes: Massachusetts (effective September 24, 2019), Maryland (February 6, 2020), New Jersey (April 20, 2020), and New York (September 19, 2019).[1,2] The control group comprised 36 states without such policies: Alabama, Arizona, Arkansas, California, Colorado, Connecticut, Florida, Georgia, Hawaii, Illinois, Indiana, Iowa, Kentucky, Louisiana, Maine, Michigan, Minnesota, Mississippi, Missouri, Montana, Nebraska, Nevada, New Hampshire, North Carolina, Ohio, Oklahoma, Oregon, Pennsylvania, South Carolina, Tennessee, Texas, Utah, Virginia, Washington, West Virginia, and Wisconsin.

Identification of study sample (never e-cigarette users)

We identified PATH study participants who had never used e-cigarettes at baseline wave interview using the marker variable "*Respondent has ever used an e-cigarette as of the last wave he/she participated in*" from the PATH Restricted-Use File (ICPSR 36231.v39) and Special Collection Restricted-Use File (ICPSR 37519.v10). Never-users were defined as participants with a value of 2 ("No") for this marker variable, indicating they had never used an e-cigarette at the time of their last wave interview. During special data collection (Waves 4.5 and 5.5), the PATH study only conducted interviews with specific age groups: Wave 4.5 collected data from participants aged 12–17, and Wave 5.5 collected data from participants aged 13–19. Therefore, study sample identified in these special data collection waves only included youth and young adults.

Baseline Wave	Participant	Variable	Data File
4	Adult & youth	R04M_EVR_ECIG	ICPSR 36231
4.5	Youth	X04M_EVR_EPRODS	ICPSR 37519
5	Adult & youth	R05M_EVR_ECIG	ICPSR 36231
5.5	Adult & youth	X05M_EVR_EPRODS	ICPSR 37519
6	Adult	R06R_A_EVR_EPRODS	ICPSR 36231
6	Youth	R06R_Y_EVR_EPRODS	ICPSR 36231

Outcome variable

For each e-cigarette naïve participant identified at baseline, we assessed whether they initiated e-cigarette use between baseline and follow-up wave interviews using a "never to ever" indicator derived by the PATH study. For the youth sample identified at Wave 4, we used X04R_Y_NEW_EPRODS ("*Wave 4.5 Youth Never to Ever Electronic Nicotine Product User*") at

© 2026 Lin M-Y et al. *JAMA Network Open*.

Wave 4.5 to determine vaping initiation. Participants with a value of 1 ("Yes") were coded as having initiated e-cigarette use at follow-up, indicating they started using electronic nicotine products between Wave 4 and Wave 4.5. For the adult sample identified at Wave 4, the subsequent wave was Wave 5 (as Wave 4.5 only collected data from youth); we used R05R_A_NEW_EPRODS ("*Wave 5 Adult Never to Ever Electronic Nicotine Product User*") to determine e-cigarette initiation. The following table lists variables used to code outcomes for each follow-up wave.

Follow-up Wave	Participant	Variable	Data File
4.5	Youth	X04R_Y_NEW_EPRODS	ICPSR 37519
5	Adult	R05R_A_NEW_EPRODS	ICPSR 36231
5	Youth	R05R_Y_NEW_EPRODS	ICPSR 36231
5.5	Adult	X05R_A_NEW_EPRODS	ICPSR 37519
5.5	Youth	X05R_Y_NEW_EPRODS	ICPSR 37519
6	Adult	T05R_A_NEW_EPRODS	ICPSR 36231
6	Youth	R06R_A_NEW_EPRODS	ICPSR 36231
7	Adult	R07R_A_NEW_EPRODS	ICPSR 36231
7	Youth	R07R_Y_NEW_EPRODS	ICPSR 36231

Covariates

Socio-demographics

For each study sample identified at baseline waves, we extracted relevant sociodemographic variables from PATH Restricted Use File, including age, sex, race/ethnicity, household income, parental education or educational attainment, school grades (youth only), and sexual minority status. The following description uses Wave 4 data fields as an example to illustrate variable construction across all waves.

Age groups were constructed from derived yearly age fields (R04R_A_AGE for adults, R04R_Y_AGE for youth). Biological sex was determined using R04R_A_SEX for adults and R04R_Y_SEX for youth. Race and ethnicity were assessed using race variables (R04R_A_RACE for adults, R04R_Y_RACE for youth) combined with Hispanic ethnicity indicators (R04R_A_HISP for adults, R04R_Y_HISP for youth).

Household income was measured using R04_AM0030 for adults and R04_PM0130 for youth, then categorized into five levels: less than $10,000, $10,000-$24,999, $25,000-$49,999, $50,000-$99,999, and $100,000 or more. Educational variables differed by sample type, with parental education captured for youth participants (R04_PM0001) and individual educational attainment recorded for adults (R04_AM0018). Both education measures were categorized into

© 2026 Lin M-Y et al. *JAMA Network Open*.

five levels: less than high school or some high school without diploma, GED, high school diploma, some college or associate degree, and bachelor's degree or higher.

Grade level was available exclusively for youth participants (R04_YM0018) and categorized from sixth grade through eleventh grade, with an additional "other" category. Sexual minority status was determined from sexual orientation responses (R04_AM0063 for adults, R04_YM0063 for youth), coded as non-sexual minority for "straight" responses and sexual minority for "lesbian or gay," "bisexual," or "something else" responses.

Psychosocial Distress

Psychosocial distress was measured using the Global Appraisal of Individual Needs–Short Screener (GAIN-SS), which assesses four internalizing symptoms and seven externalizing symptoms. Participants were coded as experiencing a specific symptom if they reported having the symptom during the "past month." The following description uses Wave 4 data fields to illustrate variable construction across all waves.

The four *internalizing symptoms* were assessed through the following items: Depression was measured by asking about "significant problems with feeling very trapped, lonely, sad, blue, depressed or hopeless about the future" (R04_AX0161 for adults, R04_YX0161 for youth). Sleep disturbances were captured through questions about "significant problems with sleep trouble such as bad dreams, sleeping restlessly or falling asleep during the day" (R04_AX0162 for adults, R04_YX0162 for youth). Anxiety was assessed by asking about "significant problems with feeling very anxious, nervous, tense, scared, panicked or like something bad was going to happen" (R04_AX0163 for adults, R04_YX0163 for youth). Emotional distress was measured through questions about "becoming very distressed and upset when something reminded you of the past" (R04_AX0164 for adults, R04_YX0164 for youth).

The seven *externalizing symptoms* were indicated by responses of "past month" to corresponding survey items. Lying or conning behavior was assessed through questions about lying or conning "to get things you wanted or to avoid having to do something" (R04_AX0165 for adults, R04_YX0165 for youth). Attention difficulties were measured by asking about having "a hard time paying attention at school, work or home" (R04_AX0166 for adults, R04_YX0166 for youth). Listening problems were captured through questions about having "a hard time listening to instructions at school, work or home" (R04_AX0167 for adults, R04_YX0167 for youth). Bullying behavior was assessed by asking about being "a bully or threatened other people" (R04_AX0168 for adults, R04_YX0168 for youth). Physical aggression was measured through questions about starting "physical fights with other people" (R04_AX0169 for adults,

© 2026 Lin M-Y et al. *JAMA Network Open*.

R04_YX0169 for youth). Restlessness was captured by asking about feeling "restless or the need to run around or climb on things" (R04_AX0250 for adults, R04_YX0250 for youth). Impulsivity was assessed through questions about giving "answers before the other person finished asking the question" (R04_AX0251 for adults, R04_YX0251 for youth).

State Tobacco Control Policies

We adjusted for three state-level tobacco control policies: Tobacco 21 laws, e-cigarette taxes, and tobacco control program spending. For Tobacco 21 laws, we identified whether a state or federal law prohibiting tobacco sales to individuals under age 21 was in effect at the start of each quarter, using effective dates from the Preventing Tobacco Addiction Foundation.[3] This information was used to generate a quarterly binary indicator (0 = no law; 1 = law in effect). We coded a quarterly binary variable indicating the presence of e-cigarette taxes in a given state at the beginning of the quarter, using data reported by Cotti et al.[4] Tobacco control spending was measured as a percentage of CDC-recommended funding levels, using data from the Campaign for Tobacco-Free Kids[5] and categorized as: ≤25%, 26–50%, 51–75%, and >75%.

State Economic Climate

We measured state-level economic climate using annual poverty rates (the percentage of the population living in poverty)[6] and quarterly unemployment rates.[7]

Emergency Events

Emergency events included the e-cigarette or vaping product use-associated lung injury (EVALI) outbreak and the COVID-19 pandemic. For the EVALI outbreak, we aggregated state-level hospitalized EVALI cases from June 2019 to February 2020 using CDC data.[8] EVALI case counts were coded as zero before June 2019 and remained constant after February 2020.[9] We categorized state-level EVALI cases into seven groups: 0, 1-9, 10-49, 50-99, 100-149, 150-199, and 200-249 cases. For the COVID-19 pandemic, we assessed state-level pandemic impact using the logarithm of cumulative COVID-19 deaths, derived from CDC's Weekly COVID-19 Cases and Deaths data[10] for each quarter. The logarithmic transformation was applied to avoid assuming a linear relationship between COVID-19 pandemic severity and the odds of e-cigarette initiation, allowing for a more flexible modeling approach that better captures the potential non-linear effects of pandemic impact on behavioral outcomes. We also adjusted for whether the follow-up interview was conducted during a state closure for the COVID-19 pandemic.[11]

Model Specification

© 2026 Lin M-Y et al. *JAMA Network Open*.

To estimate the effect of flavored e-cigarette sales bans, we applied Gardner's two-stage difference-in-differences approach,[12] which accounts for staggered policy adoption and addresses potential bias in traditional two-way fixed effects models. In the first stage, we estimated the following linear probability model:

$$Y_{i,s,t} = \beta_0 + \beta_1 \, Ban_{s,t} + \gamma \mathbb{X}_{i,s,t} + \delta \mathbb{Z}_{s,t} + \pi_s + \theta_t + \varepsilon_{i,s,t}.$$

where $Y_{i,s,t}$ is the outcome for individual *i* in state *s* at year-quarter *t*. The key independent variable, $Ban_{s,t}$, equals 1 if a flavored e-cigarette sales ban was in effect in state *s* at the beginning of quarter *t*, and 0 otherwise. $\mathbb{X}_{i,s,t}$ and $\mathbb{Z}_{s,t}$ are vectors of individual- and state-level covariates described above, respectively. We included state fixed effects (π_s) to control for time-invariant state characteristics (e.g., social norms around tobacco use) and time fixed effects (θ_t) to account for nationwide policy shifts (e.g., federal flavored e-cigarette regulation actions in 2020), seasonal trends, and changes in PATH survey questions across waves. In the second stage, we aggregated group-time-specific treatment effects to obtain an unbiased estimate of the average treatment effect on the treated. Analyses used sampling weights to account for the complex survey design,[13] and standard errors were clustered at the state level and estimated using the Huber-White robust variance estimator.[14,15]

We implemented Gardner's two-stage difference-in-differences analysis using the Stata command *did2s*.[12,16] To assess the parallel-trends assumption, we estimated event-study models including leads and lags of the flavor-ban indicator, controlling for state and quarter fixed effects as well as the covariates used in the two-stage DiD analysis. We plotted the estimated pre-policy coefficients with 95% confidence intervals and performed joint tests to assess the equality of pre-policy coefficients.

Sensitivity Analysis

We conducted several sensitivity analyses to assess the robustness of our results. First, we excluded one treatment state at a time (leave-one-out analysis) to ensure no single state drove the findings. Second, we extended the study period to 2016 to capture earlier trends in flavored e-cigarette use. Third, we excluded Washington and Montana, which enacted temporary emergency flavor bans during the 2019 EVALI outbreak,[17,18] and Utah, which restricted flavored e-cigarette sales to tobacco specialty retailers.[1]

We conducted additional sensitivity analyses to test the robustness of our findings. First, we excluded bordering states to evaluate potential spillover effects from cross-state purchasing. Second, we excluded California and Minnesota, where many local jurisdictions implemented

© 2026 Lin M-Y et al. *JAMA Network Open*.

flavor restrictions during the study period, to minimize confounding from unmeasured local policies. Third, we restricted analyses to participants who remained in the same state between survey waves to address possible exposure misclassification. Fourth, we adjusted for state smoke-free air policies to account for broader tobacco control environments. We measured these laws by the share of the population covered by indoor smoking and vaping restrictions, using data from the American Nonsmokers Rights Foundation.[19] Finally, we re-estimated models using wild cluster bootstrap procedures to ensure valid inference given the small number of treated clusters. Results from all sensitivity analyses were consistent with the main findings, confirming the robustness of our conclusions (**eTable 2**).

© 2026 Lin M-Y et al. *JAMA Network Open*.

eFigure 1. Parallel Trend Assumption Test: Main Analyses across Three Age Groups and Subgroup Analyses Limited to Young Adults (18-24)

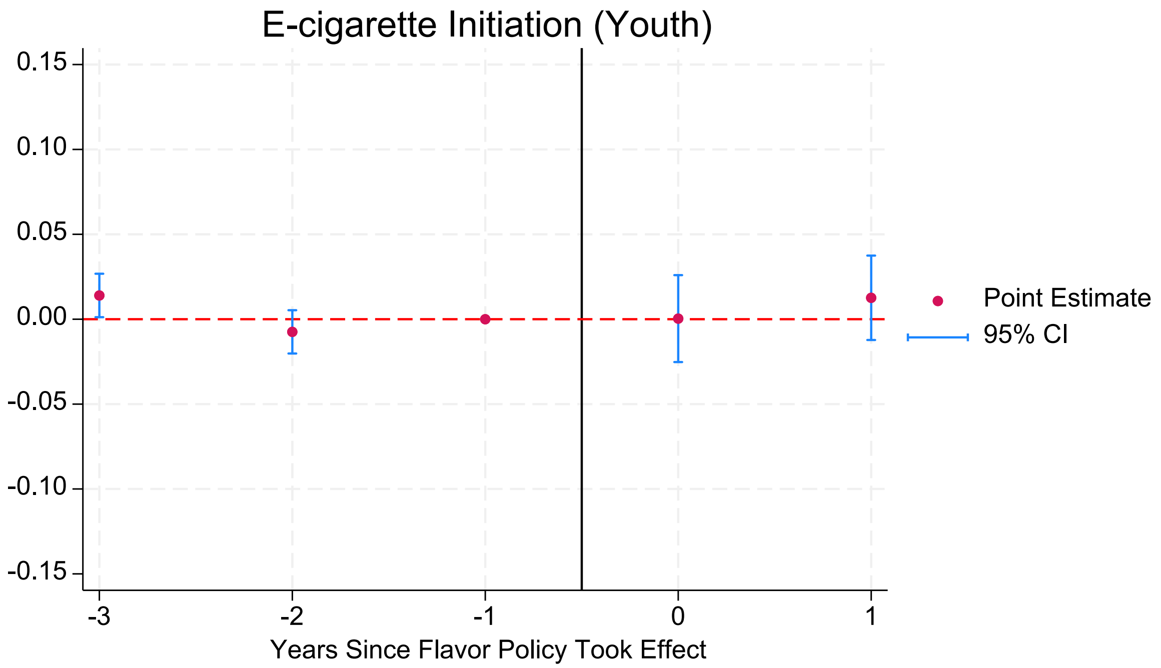

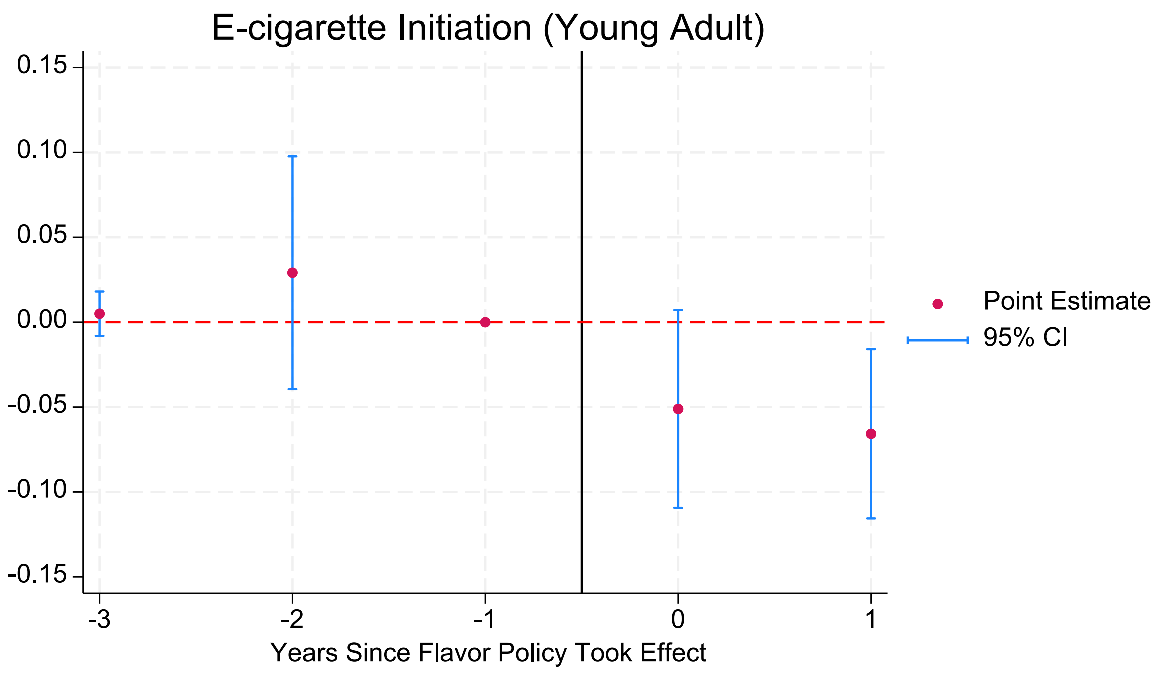

© 2026 Lin M-Y et al. *JAMA Network Open*.

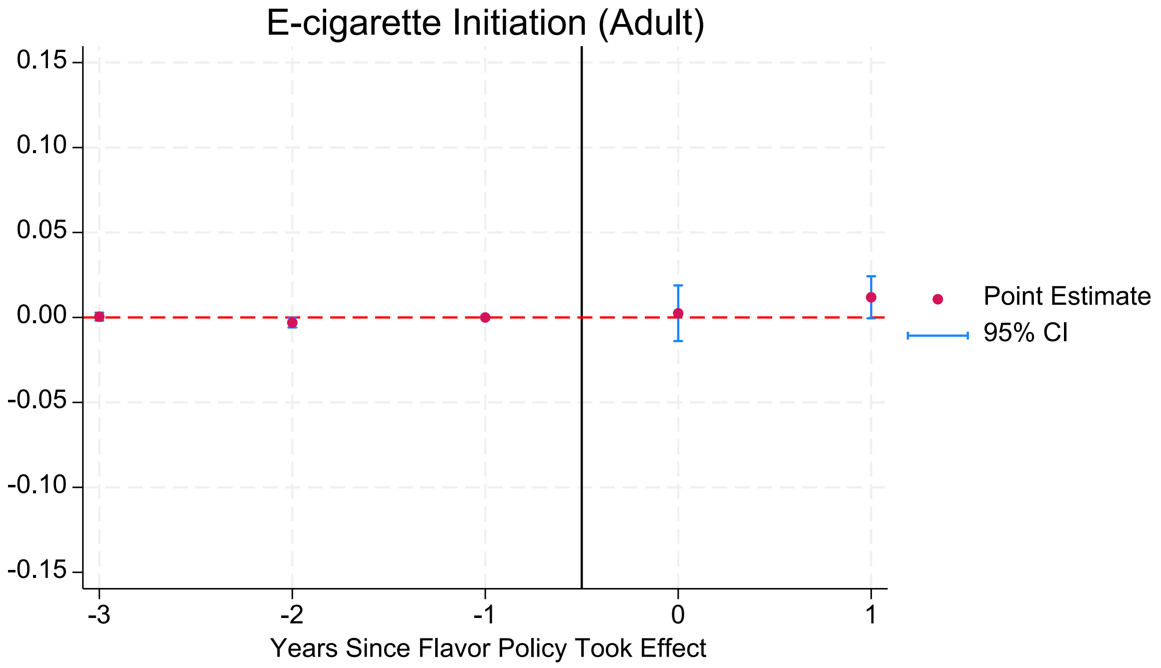

© 2026 Lin M-Y et al. *JAMA Network Open*.

eTable 1. Event Study Analysis of Statewide Flavored E-Cigarette Sales Bans and E-cigarette Initiation

	Youth (12-17)			Young Adult (18-24)			Adult (25+)		
Event time (years)[a]	Coefficient	95% CI	p-value	Coefficient	95% CI	p-value	Coefficient	95% CI	p-value
≤ -3	0.014	[0.118, 2.679]	0.03	0.005	[-0.808, 1.806]	0.45	0.000	[-0.192, 0.282]	0.71
-2	-0.007	[-2.021, 0.529]	0.25	0.029	[-3.944, 9.768]	0.41	-0.003	[-0.588, -0.019]	0.04
-1 (reference)	-	-	-	-	-	-	-	-	-
0	0.000	[-2.525, 2.696]	0.98	-0.051	[-10.935, 0.717]	0.09	0.002	[-1.390, 1.884]	0.77
≥ 1	0.013	[-1.224, 3.746]	0.32	-0.066	[-11.558, -1.591]	0.01	0.012	[-0.048, 2.425]	0.06
Test for equality of pre-treatment coefficients[b] (p-value)		0.098			0.199			0.109	

Notes:

[a] Coefficients indicate percentage-point changes relative to one year before policy implementation. Standard errors are clustered at the state level.

[b] Equality of pre-treatment coefficients was assessed using a post-estimation Wald test.

© 2026 Lin M-Y et al. *JAMA Network Open*.

eTable 2. Sensitivity Analysis: Impact of Statewide Flavored E-Cigarette Sales Bans on E-cigarette Initiation among Young Adults (18-24)

	Treatment States[a] (n=4)			Control States (n=36)			Differential Change			
	pre-ban[b] initiation rate	post-ban[b] initiation rate	pre-post change (pp)	pre-ban initiation rate	post-ban initiation rate	pre-post change (pp)	Unadjusted DiD (pp)	p-value	Adjusted DiD (pp) [95% CI]	p-value
Main analysis	10.8%	5.7%	-5.12	8.6%	7.2%	-1.40	-3.72	0.05	-6.05 [-11.21, -0.90]	0.02
Drop one treatment state										
MA	10.2%	6.1%	-4.14	8.6%	7.2%	-1.40	-2.74	0.17	-5.57 [-11.60, 0.45]	0.07
MD	10.1%	6.1%	-4.04	8.6%	7.2%	-1.40	-2.64	0.21	-5.79 [-11.65, 0.07]	0.05
NJ	11.3%	5.4%	-5.88	8.6%	7.2%	-1.40	-4.48	0.04	-7.97 [-12.40, -3.53]	<0.001
NY	12.4%	5.2%	-7.14	8.6%	7.2%	-1.40	-5.74	0.02	-5.83 [-11.46, -0.19]	0.04
Include 2016 Q1 to Q4	8.9%	5.7%	-3.16	7.7%	7.2%	-0.51	-2.65	0.09	-4.23 [-9.01, 0.54]	0.08
Drop three control states: WA, MT & UT[c]	10.9%	5.7%	-5.12	8.7%	7.3%	-1.44	-3.68	0.05	-5.99 [-11.56, -0.43]	0.04
Drop bordering states[d]	10.8%	5.7%	-5.12	8.3%	7.1%	-1.25	-3.87	0.04	-5.59 [-10.91, -0.27]	0.04
Drop CA & MN[e]	10.8%	5.7%	-5.12	8.6%	7.5%	-1.13	-3.99	0.04	-17.09 [-30.88, -3.31]	0.02
Drop cross-state movers[f]	10.4%	5.7%	-4.72	8.4%	7.1%	-1.34	-3.38	0.07	-4.93 [-9.93, -0.08]	0.04
Adjust for state smoke-free policies[g]	10.8%	5.7%	-5.12	8.6%	7.2%	-1.40	-3.72	0.05	-6.29 [-11.46, -1.13]	0.02
Wild cluster bootstrap[h]	10.8%	5.7%	-5.12	8.6%	7.2%	-1.40	-3.72	0.05	-6.05 [-11.44, -0.33]	<0.001

pp: percentage point. DiD: difference-in-differences.

Notes:

[a] Treatment states include Massachusetts (MA), Maryland (MD), New Jersey (NJ), and New York (NY).

[b] The pre-ban period spanned from 2017 Q1 to 2019 Q3 for MA & NY, from 2017 Q1 to 2020 Q1 for MD, and from 2017 Q1 to 2020 Q2 for NJ. The post-ban period extended from 2019 Q4 to 2021 Q4 for MA & NY, from 2020 Q2 to 2021 Q4 for MD, and from 2020 Q3 to 2021 Q4 for NJ.

[c] Washington[18] and Montana[17] both implemented temporary, emergency statewide bans on the sale of flavored e-cigarettes in response to the 2019 EVALI (vaping-related lung injury) crisis. Utah limited the sale of flavored e-cigarettes to retail tobacco specialty businesses (tobacco/vape shops) before stateside flavor ban took effect in January 2025.[20]

[d] Six bordering states (NH, VT, CT, PA, WV, VA) were excluded from the control group.

[e] Many cities and counties in California and Minnesota implemented local policy restricting sales of flavored e-cigarettes during the study period.

[f] Participants who moved across states between baseline and follow-up interviews were excluded.

[g] The two-stage DiD model was further adjusted for the share of state population covered by local or state smoke-free laws, including smoking and vaping.

[h] The two-stage DiD model with standard errors calculated by wild cluster bootstrap.

© 2026 Lin M-Y et al. *JAMA Network Open*.

eTable 3. Subgroup Analysis: Impact of Statewide Flavored E-Cigarette Sales Bans on E-cigarette Initiation among Youth (12-17)

	Treatment States[a] (n=4)			Control States (n=36)			Differential Change			
	pre-ban[b] initiation rate	post-ban[b] initiation rate	pre-post change (pp)	pre-ban initiation rate	post-ban initiation rate	pre-post change (pp)	Unadjusted DiD (pp)	p-value	Adjusted DiD (pp) [95% CI]	p-value
Main analysis	8.9%	6.3%	-2.60	9.5%	6.4%	-3.01	0.41	0.73	-0.56 [-1.70, 2.83]	0.63
Sex										
Female	8.6%	6.8%	-1.83	9.9%	7.1%	-2.88	1.05	0.56	-0.63 [-2.39, 3.65]	0.68
Male	9.1%	5.8%	-3.36	9.0%	5.9%	-3.14	-0.22	0.89	-0.50 [-1.60, 2.60]	0.64
Race/ethnicity										
Hispanic	7.6%	6.9%	-0.72	7.3%	6.0%	-1.28	0.56	0.83	2.60 [-0.03, 5.23]	0.05
Non-Hispanic Black	4.8%	8.1%	3.30	5.9%	5.4%	-0.50	3.80	0.27	5.57 [2.10, 9.05]	0.002
Non-Hispanic White	11.8%	7.1%	-4.70	12.0%	7.4%	-4.60	-0.10	0.97	-0.87 [-3.68, 1.94]	0.55
Others	7.2%	2.0%	-5.17	7.3%	5.0%	-2.38	-2.79	0.13	-2.57 [-4.68, -0.46]	0.005
Household Income										
<50k	8.8%	6.1%	-2.68	8.3%	7.0%	-1.29	-1.39	0.54	1.96 [0.05, 3.87]	0.04
50k+	9.1%	6.8%	-2.29	10.4%	6.3%	-4.13	1.84	0.22	0.44 [-2.08, 2.96]	0.73
Unknown	5.8%	0.0%	-5.83	7.5%	4.3%	-3.20	-2.63	0.35	-3.96 [-6.74, -1.18]	0.005
Psychosocial Distress[c]										
Any Internalizing Symptom	10.5%	7.7%	-2.82	11.5%	8.0%	-3.49	0.67	0.72	0.13 [-4.61, 4.87]	0.96
No Internalizing Symptom	7.2%	4.8%	-2.36	7.5%	5.1%	-2.39	0.03	0.99	1.26 [-1.42, 3.93]	0.36
Any Externalizing Symptom	9.5%	8.5%	-1.06	11.6%	8.0%	-3.60	2.54	0.15	0.84 [-2.30, 3.98]	0.60
No Externalizing Symptom	8.0%	3.3%	-4.73	6.8%	4.4%	-2.33	-2.40	0.11	0.52 [-1.59, 2.63]	0.63
Sexual Minority Status[d]										
LGB	9.6%	9.6%	-0.01	13.9%	10.4%	-3.55	3.54	0.39	-0.17 [-9.09, 8.76]	0.97
Non-LGB	11.4%	5.5%	-5.87	12.0%	6.6%	-5.35	-0.52	0.73	0.83 [-2.47, 4.14]	0.62

pp: percentage point. LGB: lesbian, gay, and bisexual people.

Notes:

[a] Treatment states include Massachusetts (MA), Maryland (MD), New Jersey (NJ), and New York (NY).

[b] The pre-ban period spanned from 2017 Q1 to 2019 Q3 for mA & NY, from 2017 Q1 to 2020 Q1 for MD, and from 2017 Q1 to 2020 Q2 for NJ. The post-ban period extended from 2019 Q4 to 2021 Q4 for MA & NY, from 2020 Q2 to 2021 Q4 for MD, and from 2020 Q3 to 2021 Q4 for NJ.

[c] Psychosocial distress was measured by the Global Appraisal of Individual Needs–Short Screener, including 4 internalizing symptoms (depression, sleep disturbances, anxiety, emotional distress) and 7 externalizing symptoms (lying/conning, attention difficulties, listening issues, bullying, fights, restlessness, impulsivity).

© 2026 Lin M-Y et al. *JAMA Network Open*.

[d] Analyses stratified by sexual minority status were limited to youth aged 14-17, as the sexual orientation question was only administered to this age group.

eTable 4. Impact of State- Level Flavored E-Cigarette Sales Bans on Initiation among Young Adults (18-24), Stratified by Age (18-20 vs. 21-24)

	Treatment States[a] (n=4)			Control States (n=36)			Differential Change			
	pre-ban[b] initiation rate	post-ban[b] initiation rate	pre-post change (pp)	pre-ban initiation rate	post-ban initiation rate	pre-post change (pp)	Unadjusted DiD (pp)	p-value	Adjusted DiD (pp) [95% CI]	p-value
Young Adults (18-24)	10.9%	5.7%	-5.12	8.6%	7.2%	-1.40	-3.72	0.05	-6.05 [-11.21, -0.90]	0.02
18–20	12.4%	7.0%	-5.44	12.6%	8.5%	-4.11	-1.33	0.59	-2.51 [-6.95, 1.92]	0.27
21–24	9.8%	3.8%	-6.04	5.2%	4.9%	-0.31	-5.73	0.03	-10.79 [-20.77, -0.80]	0.03

pp: percentage point. DiD: difference-in-differences.

© 2026 Lin M-Y et al. *JAMA Network Open*.

REFERENCE

1. Bach L. States & Localities That Have Restricted the Sale of Flavored Tobacco Products. Published online May 14, 2025. Accessed June 16, 2025. https://assets.tobaccofreekids.org/factsheets/0398.pdf

2. U.S. Sales Restrictions on Flavored Tobacco Products. Published online June 2025. Accessed June 16, 2025. https://www.publichealthlawcenter.org/sites/default/files/resources/US-sales-restrictions-flavored-tobacco-products.pdf

3. Tobacco 21 Grade Cards. Tobacco 21. Accessed June 17, 2025. https://tobacco21.org/state-grades/

4. Cotti C, Nesson E, Pesko MF, Phillips S. Standardising the measurement of e-cigarette tax rates in the USA (2nd edition), 2010-2023. *Tob Control*. Published online November 27, 2024:tc-2024-058618. doi:10.1136/tc-2024-058618

5. History of spending for state tobacco prevention programs. 2023. Accessed November 1, 3AD. https://www.google.com/url?sa=t&rct=j&q=&esrc=s&source=web&cd=&ved=2ahUKEwi9zrLXk6v9AhUKMlkFHTkcAhsQFnoECBUQAQ&url=https%3A%2F%2Fwww.tobaccofreekids.org%2Fassets%2Ffactsheets%2F0209.pdf&usg=AOvVaw3YB8lTvIhbhxj2yTcX43DI

6. Bureau UC. Small Area Income and Poverty Estimates (SAIPE) Program. Census.gov. Accessed October 17, 2024. https://www.census.gov/programs-surveys/saipe.html

7. US Bureau of Labor Statistics. States and selected areas: employment status of the civilian noninstitutional population. Accessed October 17, 2024. https://www.bls.gov/web/laus/ststdnsadata.txt

8. Health CO on S and. Outbreak of Lung Injury Associated with the Use of E-Cigarette, or Vaping, Products. Centers for Disease Control and Prevention-CDC Archive. August 3, 2021. Accessed June 16, 2025. https://archive.cdc.gov/www_cdc_gov/tobacco/basic_information/e-cigarettes/severe-lung-disease.html

9. Ali FRM, Vallone D, Seaman EL, et al. Evaluation of Statewide Restrictions on Flavored e-Cigarette Sales in the US From 2014 to 2020. *JAMA Netw Open*. 2022;5(2):e2147813. doi:10.1001/jamanetworkopen.2021.47813

10. Weekly United States COVID-19 Cases and Deaths by State - ARCHIVED. Centers for Disease Control and Prevention. Accessed June 16, 2025. https://data.cdc.gov/Case-Surveillance/Weekly-United-States-COVID-19-Cases-and-Deaths-by-/pwn4-m3yp/about_data

11. Dasgupta S, Kassem AM, Sunshine G, et al. Differences in rapid increases in county-level COVID-19 incidence by implementation of statewide closures and mask mandates - United States, June 1-September 30, 2020. *Ann Epidemiol*. 2021;57:46-53. doi:10.1016/j.annepidem.2021.02.006

12. Gardner J, Thakral N, Tô LT, Yap L. Two-Stage Differences in Differences. https://www.bu.edu/econ/files/2024/07/two-stage-differences-in-differences.pdf

© 2026 Lin M-Y et al. *JAMA Network Open*.

13. Underwood JM, Brener N, Thornton J, et al. Overview and Methods for the Youth Risk Behavior Surveillance System - United States, 2019. *MMWR Suppl*. 2020;69(1):1-10. doi:10.15585/mmwr.su6901a1

14. Huber PJ. The behavior of maximum likelihood estimates under nonstandard conditions. In: Fifth Berkeley Symposium on Mathematical Statistics and Probability. University of California Press; 1967:221-233. https://projecteuclid.org/euclid.bsmsp/1200512988

15. White H. A Heteroskedasticity-Consistent Covariance Matrix Estimator and a Direct Test for Heteroskedasticity. *Econometrica*. 1980;48(4):817-838. doi:10.2307/1912934

16. Butts K. DID2S: Stata module to estimate a TWFE model using the two-stage difference-in-differences approach. *Stat Softw Compon*. Published online April 28, 2023. Accessed October 28, 2025. https://ideas.repec.org//c/boc/bocode/s458951.html

17. O'Brien EF. Judge Rules In Favor Of Montana's Temporary Vape Ban. Montana Public Radio. December 19, 2019. Accessed June 16, 2025. https://www.mtpr.org/montana-news/2019-12-18/judge-rules-in-favor-of-montanas-temporary-vape-ban

18. sdcadmin. Washington state lawmakers back down from flavored vape ban. Sen. Manka Dhingra. February 5, 2020. Accessed June 16, 2025. https://senatedemocrats.wa.gov/dhingra/2020/02/05/washington-state-lawmakers-back-down-from-flavored-vape-ban/

19. Percent of U.S. State Populations Covered by 100% Smokefree Air Laws. American Nonsmokers' Rights Foundation. https://no-smoke.org/wp-content/uploads/pdf/percentstatepops.pdf

20. Miller S. Utah's flavored vape ban — and lawsuit — could be out the door with new bill. KUER. February 20, 2025. Accessed October 27, 2025. https://www.kuer.org/politics-government/2025-02-20/utahs-flavored-vape-ban-and-lawsuit-could-be-out-the-door-with-new-bill

© 2026 Lin M-Y et al. *JAMA Network Open*.
