# Supplementary material for: State-Level Flavored E-Cigarette Bans and Initiation Rates Among Youths and Adults
Source: JAMA Netw Open. 2026 Jan 5;9(1):e2551744. doi: 10.1001/jamanetworkopen.2025.51744 (PMC12771250; doi:10.1001/jamanetworkopen.2025.51744)
Supplement: Supplement 2. — Data Sharing Statement [file jamanetwopen-e2551744-s002.pdf]

Data Sharing Statement

Lin. State-Level Flavored E-Cigarette Bans and Initiation Rates Among Youths and Adults. *JAMA Netw Open*. Published online January 5, 2026. doi:10.1001/jamanetworkopen.2025.51744

Data

Data available: No

Additional Information

Explanation for why data not available: The PATH survey is a publicly available dataset; however, the state identifying information is restricted access. Interested parties can obtain the data from https://www.icpsr.umich.edu/web/NAHDAP/series/606 .
